# Geographic selection bias of occurrence data influences transferability of invasive *Hydrilla verticillata* distribution models

**DOI:** 10.1002/ece3.1120

**Published:** 2014-05-26

**Authors:** Matthew A Barnes, Christopher L Jerde, Marion E Wittmann, W Lindsay Chadderton, Jianqing Ding, Jialiang Zhang, Matthew Purcell, Milan Budhathoki, David M Lodge

**Affiliations:** 1 Environmental Change Initiative, University of Notre DameNotre Dame, Indiana; 2 The Nature ConservancySouth Bend, Indiana; 3 Wuhan Botanical Garden, Chinese Academy of SciencesWuhan, China; 4 Agricultural Research Service, Australian Biological Control Laboratory, United States Department of AgricultureBrisbane, Queensland, Australia; 5 Center for Research Computing, University of Notre DameNotre Dame, Indiana

**Keywords:** Aquatic macrophyte, biological invasion, habitat model, maximum entropy, niche model, prediction, spatial bias

## Abstract

Due to socioeconomic differences, the accuracy and extent of reporting on the occurrence of native species differs among countries, which can impact the performance of species distribution models. We assessed the importance of geographical biases in occurrence data on model performance using *Hydrilla verticillata* as a case study. We used Maxent to predict potential North American distribution of the aquatic invasive macrophyte based upon training data from its native range. We produced a model using all available native range occurrence data, then explored the change in model performance produced by omitting subsets of training data based on political boundaries. We also compared those results with models trained on data from which a random sample of occurrence data was omitted from across the native range. Although most models accurately predicted the occurrence of *H. verticillata* in North America (AUC > 0.7600), data omissions influenced model predictions. Omitting data based on political boundaries resulted in larger shifts in model accuracy than omitting randomly selected occurrence data. For well-documented species like *H. verticillata*, missing records from single countries or ecoregions may minimally influence model predictions, but for species with fewer documented occurrences or poorly understood ranges, geographic biases could misguide predictions. Regardless of focal species, we recommend that future species distribution modeling efforts begin with a reflection on potential spatial biases of available occurrence data. Improved biodiversity surveillance and reporting will provide benefit not only in invaded ranges but also within under-reported and unexplored native ranges.

## Introduction

Globalization of commerce and travel has increased the rates of introduction of nonindigenous species throughout the world (Hulme 2009). Understanding the future distribution of potentially harmful species represents a key step in minimizing the damages associated with these introductions. The use of models to predict potential species ranges has become common in the study and management of biological invasions (Thuiller et al. 2005). In general, such models correlate known species occurrences with local environmental data, such as climatic patterns, to characterize the niche and predict the likelihood that species will find suitable habitat for establishment in new locations. Over the last decade, improvements in computing power and increased data availability have contributed to the development of different methods for predicting species ranges, collectively referred to as species distribution models (reviewed in Franklin 2009).

Distribution modeling of nonindigenous species presents several challenges, especially when spatial gaps in occurrence records exist. For instance, predicting suitable habitat based on native range occurrences can require extrapolation to novel environmental conditions, and species distribution modeling methods differ considerably in their ability to perform such extrapolations (Elith and Graham 2009). The ability to use models developed in one region to predict species distribution in another is referred to as model transferability and represents an active area of research (Randin et al. 2006; Peterson et al. 2007; Phillips 2008; Heikkinen et al. 2012).

Combining occurrence data from native and introduced ranges can influence model projections (Mau-Crimmins et al. 2006; Loo et al. 2007). A key assumption of species distribution modeling is that occurrence data represent populations at equilibrium with their environment (Pearman et al. 2008; Warren 2012), yet occurrence data from introduced populations may not represent the total extent of habitable space in a given landscape due to dispersal limitation or lags following an initial introduction (Broennimann and Guisan 2008; Václavíc and Meentemeyer 2009, 2012; Robinson et al. 2010). On the other hand, the attention received by the establishment of invasive species likely increases the frequency and accuracy of occurrence data reporting within introduced ranges compared to native ranges where species are not often monitored and reported. Thus, a trade-off may often exist between the use of native range data, which represents an inaccurate and incomplete portrayal of an equilibrium population, and introduced range data, which may be more thorough but depict a range still undergoing expansion.

Political boundaries represent a common spatial bias in species occurrence data; differences in investment in science and management can lead to differences in the completeness or availability of species occurrence records among countries. Ethical, esthetic, and economic incentives can all motivate governments to be proactive in biodiversity surveillance (Ehrlich and Ehrlich 1992). Furthermore, the establishment of the United Nations Environment Program and various international treaties has increased political pressure for conservation and sustainable development (Western 1989). Bioprospecting, the search for new species with pharmacological or other economic value, also provides growing motivation for species inventory and discovery (Macilwain 1998), as does the prospect of raising money by promoting ecotourism (Bookbinder et al. 1998). Regardless of motivation, the availability of biodiversity data is essential for scientists, managers, and policymakers, including those making predictions about the threat of nonindigenous invasive species around the globe (Edwards et al. 2000).

**Figure 1:**
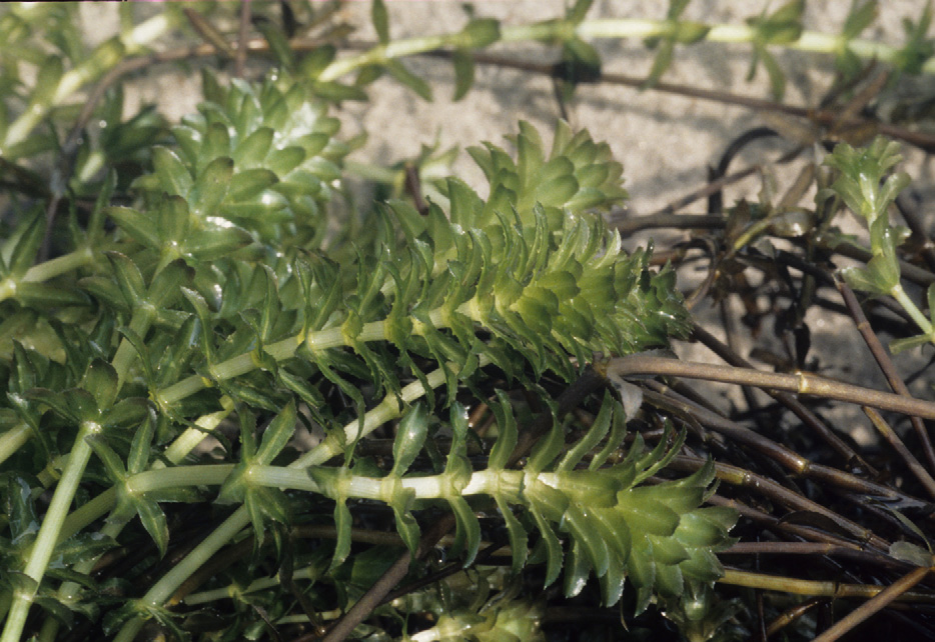
*Hydrilla verticillata* (L.f.) Royle. Photo credit: Vic Ramey, University of Florida/IFAS Center for Aquatic and Invasive Plants.

Previous research has examined the influence of occurrence data spatial bias by partitioning data randomly (Peterson et al. 2007; Phillips 2008) or reflecting other aggregation that occurs as species are detected over time (Feeley and Silman 2011; Václavíc and Meentemeyer 2012). Using the aquatic invasive macrophyte hydrilla (*Hydrilla verticillata* [L.f.] Royle; Fig. 1) as a case study, we assessed the impact of biases reflecting political boundaries in occurrence data on the transferability of species distribution models. Vegetative reproduction, broad environmental tolerance, and an aggressive growth habit have made hydrilla a formidable global invader (Langeland 1996). These traits have motivated collection of a large global occurrence data set, making hydrilla an excellent case study for exploring the performance of species distribution models. By assessing the impacts of political biases in occurrence data on the transferability of hydrilla distribution models, we can improve predictions of species habitat and provide a more confident foundation for the management of ecological reserves, surveillance of biological invasions, or efforts to better define the range of potentially threatened species.

## Methods

### Study organism

Hydrilla's native range is found in central Asia and Australia (Cook and Lüönd 1982; Buckingham and Bennett 1996). Although there is speculation that some hydrilla populations in China may represent reintroductions rather than true native populations (Benoit 2011), we consider all Chinese occurrences as representatives of the native range in the present study. Hydrilla was first detected in the United States in Florida in the 1960s (Steward et al. 1984) and has since spread across most of temperate North America. Introduced populations also occur in Central and South America, Africa, Europe, and New Zealand (Langeland 1996). Where introduced, hydrilla often has considerable impacts including reduction of flow in canals, interference with recreational activities, and displacement of native plants through competition for light and other resources (Langeland 1996).

Peterson et al. (2003) previously conducted species distribution modeling for hydrilla in the United States using the genetic algorithm for rule-set prediction (GARP; Stockwell and Peters 1999). However, recent Midwestern appearances of hydrilla in Lake Manitou, Indiana, in 2006 and a private pond in Marinette County, Wisconsin, in 2007 fall outside the potential range estimated by Peterson et al. (2003). In the present study, we used a new modeling technique and updated occurrence records to inform management of hydrilla. Management efforts will benefit from an updated demarcation of potential invasible habitat in the United States and determining previously unidentified native sources of hydrilla propagules.

### Data collection

**Figure 2:**
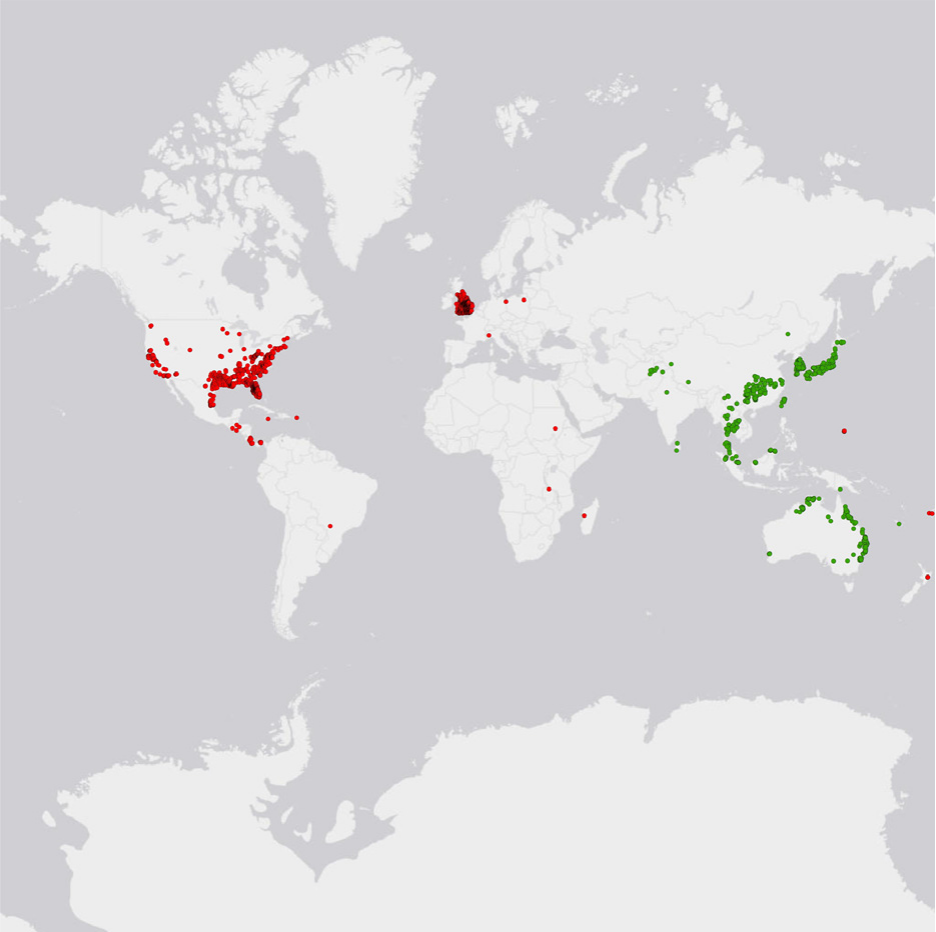
Native (*N* = 1018; green) and introduced (*N* = 3318; red) occurrences of *Hydrilla verticillata*. Appendix S2 provides list of geographic coordinates.

We accessed hydrilla occurrence data through the Global Biodiversity Information Facility online database (http://www.gbif.org accessed March 2012), the United States Geological Survey Nonindigenous Aquatic Species database (http://nas.er.usgs.gov/) and the United States Department of Agriculture National Plant Data Center PLANTS database (http://plants.usda.gov) as well as a South American occurrence reported by Anderson et al. (2005). In addition, we included data from two collection efforts along the Ohio River, USA (*N* = 33) and throughout the native range (*N* = 319) (see Appendix S1 for description of collection). Overall, we compiled 4336 total hydrilla occurrence records around the globe, including 1018 we identified as native range records (Fig. 2; see Appendix S2 for list of geographic coordinates). Each georeferenced position was verified, and error radii were assigned using the georeferencing calculator provided by MaNIS (Wieczorek et al. 2004). All localities with an uncertainty of position larger than 50 km were removed. As a first step to reduce bias that may be generated by uneven sampling effort, we rarified occurrence data before moving on to model implementation by converting the occurrence points into a Raster file with the same cell size as our environmental data, then back to a points file, resulting in a maximum of one occurrence point per cell (McDowell et al. 2014; data conversion and all further mentioned visualization performed in ArcGIS 9.3, Environmental Systems Research Institute, Redlands, California, USA). Thus, the working occurrence dataset included 323 native occurrences.

To establish the relationship between species occurrence and habitat, we selected data layers (average annual temperature, average monthly temperature, growing degree days) that had previously be used to determine hydrilla growth and establishment (Van et al. 1978; Spencer et al. 2000; Rybicki and Carter 2002). Data layers of 0.5° resolution were developed by New et al. (1999) and accessed from Atlas of the Biosphere (http://www.sage.wisc.edu/atlas/index.php). From the monthly average temperature layers, we derived two composite layers to represent warm and cold temperature extremes in each hemisphere. First, we used average monthly temperatures (December, January, and February) to represent average winter and summer temperatures for the northern and southern hemispheres, respectively. Second, we used average monthly temperatures (June, July, and August) to represent average northern hemisphere summer and average southern hemisphere winter temperatures. As an aquatic macrophyte, hydrilla establishment can occur only in aquatic habitats. However, we did not include any indicators of water availability in the environmental layers used to train our models because even in regions where standing water is not plentiful, such as the southwestern United States, hydrilla could establish if introduced into riverine backwaters, oases, or water gardens, and we did not want our model to miss suitable habitat in such areas.

### Species distribution modeling

We modeled hydrilla distribution with maximum entropy modeling (Maxent version 3.3.3k; Phillips et al. 2006). Maxent operates by comparing the probability distributions of species presence and environmental variables to develop a model that can be projected in geographic space (Phillips et al. 2006; Elith et al. 2011). Maxent provides one appropriate strategy for working with presence only data such as our hydrilla data set. Furthermore, in head-to-head comparisons, Maxent consistently outperformed other species distribution model implementations across taxa and geographic regions (Elith et al. 2006).

Because the purpose of our modeling effort was to illustrate the potential for changes in model outputs to occur in response to difference in input data rather than explicitly testing model performance (e.g., Phillips and Dudík 2008; Hijmans 2012), we generally opted for default Maxent software settings in all model runs. However, because overly complex models can limit model transferability (Warren and Seifert 2011), we conducted a regularization parameter (*β*) tuning exercise (e.g., Elith et al. 2010; Radosavljevic and Anderson 2014). We identified the default regularization parameter (*β* = 1) as most appropriate for our dataset (full details of tuning exercise available in Appendix S3). We did increase the maximum allowable model iterations to 5000 based on pilot model runs in which models took more than 500 iterations to converge on optimal solutions.

We interpreted the logistic output of each Maxent run as a probability map of hydrilla habitat suitability. In addition to visual inspection of output maps, we assessed model performance using area under the receiver operating characteristic curve (AUC) where AUC = 0.5 indicates the model predicts outcomes no better than random, and AUC ≥ 0.7 indicates strong predictive power (Hosmer and Lemeshow 2000). To calculate AUC, we used previously rarified (see “Data collection”) North American hydrilla occurrence records (*N* = 346) plus 346 randomly generated pseudo-absence records across North America (see Appendix S4). Use of the same test data across all models justified using AUC to assess relative strength across models (Jiménez-Valverde 2012).

In the first Maxent model implementation, we used all available native range hydrilla occurrences to predict suitable habitat in North America. Theoretical (Barve et al. 2011) and real-world examples (Anderson and Raza 2010) have demonstrated that the way researchers define the geographic space from which occurrences and background data are drawn can influence model predictions. Because we were interested in testing the transferability of models developed within the native range to invasible North American habitat, we limited our training landscape to only countries in which hydrilla occurrences are known as well as the countries that border them in Asia and Australia, coinciding with more general descriptions of the native range of hydrilla (Cook and Lüönd 1982; Buckingham and Bennett 1996).

To explore the influence of biasing occurrence data within the native range according to political boundaries, we developed five models using native occurrences as the training data set but omitting a different country within the native range from each model run. We conducted this exercise for each country that contributed > 5% of the total occurrence points in our native range occurrence dataset (Australia, China, Japan, South Korea, and Thailand). We judged the impact of omitting each country by calculating the absolute value of the difference between the AUC of the omission model and the model developed using all available native range data.

To explore whether political aggregation of data exacerbated the influence of data omission on model performance, we produced 10 additional models for each country in which an equally sized random sample of occurrence data were omitted from across the native range. For example, to provide comparison with the model that excluded Australia (*N* = 77 occurrences), we ran 10 models with 77 randomly selected occurrences removed from across the entire native range. We calculated AUC for each model and determined the average AUC for each set of 10 models. To avoid the statistical pitfalls of multiple comparisons, we did not perform pairwise comparisons of receiver operating characteristic curves between models (DeLong et al. 1988). Instead, we relied on graphical comparisons between median AUC of each set of 10 random-data omission models and the model with a specific country omitted.

## Results

**Figure 3:**
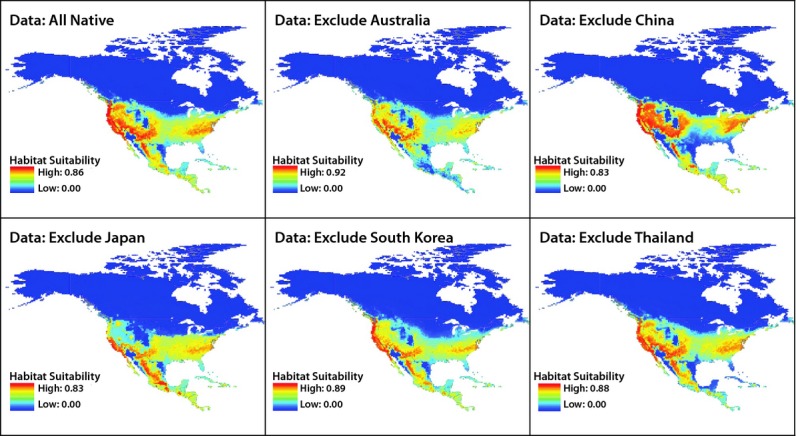
Projection of suitable *Hydrilla verticillata* habitat in North America based on separate Maxent models developed with all native range data or native range data excluding occurrences from Australia, China, Japan, South Korea, or Thailand. Shading indicates the logistic output of each model.

Multivariate environmental similarity surfaces (MESS; Elith et al. 2010) and mobility-oriented parity (MOP; Owens et al. 2013) indicated similar environmental ranges between the native and North American ranges of hydrilla with regards to the environmental layers used in this study, with the only considerable extrapolation in North America occurring in northernmost regions of Nunavut, Canada (Appendix S5). Based on model projections, much of North America appears to represent suitable habitat for hydrilla (Fig. 3). The predicted range includes many regions that are not yet known to be colonized: glacial lake districts of the upper Midwest, as well as the major reservoirs and river systems throughout much of the central and upper Midwestern United States. On the other hand, several known occurrences (e.g., in Wisconsin and Minnesota) fall outside the range projected to be suitable (Fig. 3). Little of Canada is predicted to be suitable, in contrast to the high habitat suitability of most of Mexico and Central America (Fig. 3).

Based on visual inspection, the maximum predicted extent of suitable hydrilla habitat in North America was similar between the model developed with all native range data and models excluding selected countries from the native range; however, the level of habitat suitability (our interpretation of the logistic output of each Maxent model) differed between models (Fig. 3). Most notably, compared to the model developed with all native range data, the model developed while excluding hydrilla occurrences located in China resulted in lower predicted hydrilla habitat suitability throughout the southeastern United States. Also, relative to the all native range occurrence data model, models developed while excluding data from Japan or South Korea resulted in lower predicted hydrilla habitat suitability in the central northern United States, and the model developed while excluding Japan also displayed noticeably lower predicted hydrilla habitat suitability in the United States Pacific northwest.

**Figure 4:**
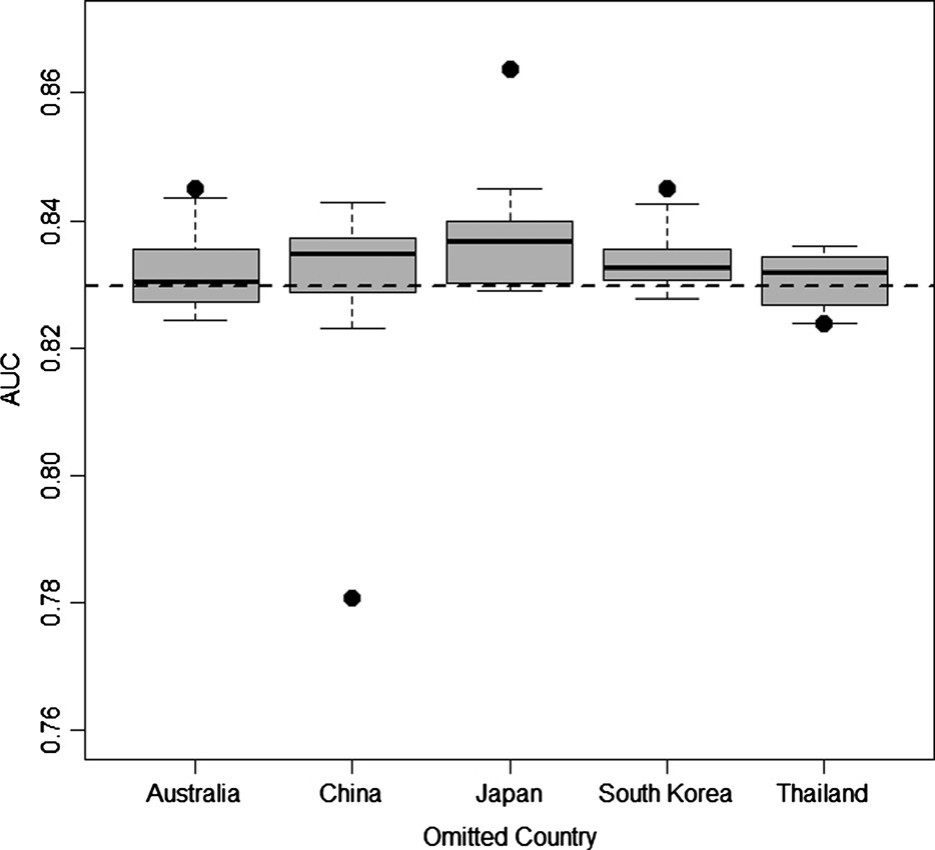
Comparison of AUCs for models used to predict *H. verticillata* occurrence in North America, but trained on different subsets of *H. verticillata* occurrence data from the native range. Dashed horizontal line indicates AUC (=0.8296) calculated for the model developed using all native range data (i.e., all countries' occurrence records included), and filled circles represent AUCs for models trained with native data from which occurrences within specific countries were excluded. Box-and-whisker plots represent 10 models developed for each country with an equal number of randomly selected data omitted from across the native range.

AUC differed among the models we developed to examine how excluding occurrence data from different political boundaries influenced predictions, but all models demonstrated considerable predictive accuracy (AUC > 0.7600) regardless of which country was excluded from the native range data set (Fig. 4). In order, the largest changes in AUC were produced by omitting China, then Japan, South Korea, Australia, and Thailand. Models developed after removal of random, rather than politically aggregated, native range occurrences also demonstrated predictive strength (AUC > 0.8000; Fig. 4). Although all models possessed strong predictive power, omitting data based on political boundaries tended to produce a larger impact on model AUC (compared to a model developed with all native range data) than did models developed with a similar number of hydrilla occurrences randomly omitted from across the native range.

## Discussion

Our analysis showed that spatial biases resulting from political gaps in data impact predictions of species distribution. In our experiments, the largest changes in model prediction strength occurred as a result of omitting China or Japan from model training, and relatively smaller effects resulted from excluding Australia, South Korea or Thailand (Fig. 4). Because geographic biases can influence predictions of species distribution models, biodiversity assessment efforts aimed at improving predictions of future range should focus on areas of conspicuous data absences, especially when such absences coincide with political delineations. For instance, in the case of hydrilla, no occurrence data were found within North Korea despite documented occurrences in nearby South Korea, Japan, and northern China (Fig. 2). Future modeling efforts would benefit from surveys in these countries to determine whether hydrilla is truly absent or the region represents an area of inadequately surveyed and underreported native habitat.

Several suggestions for removing spatial biases within occurrence data have been published including producing bias grids which prompt Maxent to weigh the importance of occurrence records inversely proportional to their proximity to neighboring occurrences (Elith et al. 2010; Kramer-Schadt et al. 2013) as well as selecting occurrence data of similar taxonomic groups that are likely to demonstrate similar bias to target occurrences as background points (e.g., Phillips et al. 2009). We explored the method of implementing bias grids in data exclusion models (Appendix S6) but found that results were consistent with models conducted without bias grids. Using occurrence data from similar species (e.g., other aquatic plant species) may result in larger bias correction if political boundaries are a major driver of spatial bias in occurrence data because such data will also likely be unaccounted for in certain countries. However, we did not test this method of bias correction in the present study.

Although somewhat counterintuitive, in some cases omitting occurrences from the training dataset improved model predictions in the invaded range. Specifically, models developed with Japan omitted from the training dataset produced notable increase in AUC relative to the model developed based on the entire native range (Fig. 4). Considering a variety of taxa and modeling techniques, previous studies have found negative relationships between model accuracy and niche breadth (Brotons et al. 2004; Tsoar et al. 2007; Evangelista et al. 2008), range size (Luoto et al. 2005; McPherson and Jetz 2007), and commonness (Franklin et al. 2009), reflecting a decrease in model ability to pinpoint suitable habitat ranges as more environmental variation is included in the training data. In our study, omitting the oceanic climate of Japan decreased the predicted suitability of habitat in the US Pacific northwestern relative to other models and increased AUC, although the general extent of the predicted range of hydrilla remained similar to other models (Fig. 3). Larger qualitative differences were observed when omitting China from the native range data, as the model predicted less suitable habitat much of the US southeastern, and AUC decreased despite the general extent of predicted hydrilla range in North America remaining similar to other models. Negotiating discrepancies between visual outputs and indicators of model performance such as AUC remains a challenge for future species distribution modeling efforts and the use of species distribution models to inform environmental management.

Increased delimitation of the native range will also help to identify sources of invasive propagules for countries implementing import screening policies to prevent introductions. False absences could lead to misplaced complacency in existing inspection regimes for international trade. The efficacy of risk-based management aimed at reducing invasions resulting from expanding international trade will increase as the accuracy and completeness of species occurrence data improve (Keller and Drake 2009). Efficient prevention of biological invasions requires engagement among nations, especially emphasizing the closing of invasion pathways at their point of origin (Meyerson and Reaser 2002). Otherwise, management of invasive species will continue to be hindered by a “weakest-link” problem in which lax regulations in one political boundary increase the overall risk of invasion (Perrings et al. 2002; Peters and Lodge 2009).

In addition to predicting the spread of invasive species under current conditions, previous species distribution modeling efforts have sought to predict shifting species ranges in response to climate change (e.g., Engler and Guisan 2009; McDowell et al. 2014). Just as geographic biases in occurrence data may hinder ability to make predictions about suitable habitat under current conditions, such biases may also lead to inaccurate predictions under future climate scenarios. In the case of hydrilla, we observed little extrapolation beyond native range environmental conditions when projecting from the native range to North America under current conditions. However, different effects of climate change between native and invaded ranges could result in greater extrapolation and decreased model performance.

Overall, much of North America, Central America, and many Caribbean islands appear to represent suitable habitat for hydrilla (Fig. 3). Our models identified far more potential habitat in the United States than previous models (Peterson et al. 2003), including more northern latitudes along the Atlantic and Pacific coasts and into the Midwest, including the southern edge of the Great Lakes. Nevertheless, the occurrence of hydrilla at sites in the upper Midwest (Wisconsin and Minnesota, Fig. 3) suggests that our model may still underestimate the potential North American range of hydrilla. At northern latitudes and high elevations, hydrilla habitat appeared to be limited by low average annual growing degree days and cooler temperatures in warmest summer months. In the southwestern United States and Mexican gulf coasts, the model predicts unsuitable hydrilla habitat, apparently reflecting constraints in the model of both high summer temperatures and temperate winters. However, numerous records for hydrilla along the Gulf of Mexico and Rio Grande provide further indication that the model underestimates hydrilla habitat suitability in North America.

Underestimations of hydrilla invasive range at its northern and southern extremes remind us that environmental factors do not necessarily tell the entire story of species distributions. A species' realized niche may also be constrained by geographic barriers to dispersal, competitors, predators, pathogens, or a limited set of available environments in natal habitats (Wiens et al. 2009; Alexander and Edwards 2010; Hill et al. 2012). Alternatively, hydrilla populations established in northern and southern extremes that are predicted as unsuitable habitats may represent evidence of niche shift (Broennimann et al. 2007; Hill et al. 2012) indicating that hydrilla in North America has either adapted to new environments or established in novel climatic zones (Alexander and Edwards 2010) as a result of human-mediated dispersal.

It is also worth noting that two hydrilla biotypes, a monoecious biotype genetically related to plants from South Korea and a dioecious biotype genetically related to plants in India (Madeira et al. 1999), occur within the North American invaded range. With the exception of Steward and Van (1987) demonstrating that the monoecious biotype is better adapted to grow in cooler temperatures and shorter photoperiods than the dioecious type, little research exists regarding the physiological differences between biotypes and their implications for invasion and ability to produce impacts. We could not differentiate between biotypes within most of our collected occurrence data and did not account for this factor in our species distribution model implementation. If more biotype information becomes available, incorporating this information into future species distribution modeling efforts may improve our ability to predict suitable hydrilla habitat in North America.

Differences between our projected models and the reality of hydrilla occurrence in North America may be the product of microscale habitat conditions not captured by our environmental layers. Furthermore, although our selection of environmental layers was motivated by research demonstrating light and temperature influence hydrilla growth and establishment (Van et al. 1978; Spencer et al. 2000; Rybicki and Carter 2002), it is likely that other factors such as water chemistry influence hydrilla establishment. Unfortunately, lack of a repository of diverse global environmental layers beyond temperature and precipitation data represents a critical limitation of species distribution modeling efforts, especially in aquatic systems.

We used hydrilla as a case study, but the conclusion that spatial biases according to political boundaries affects accuracy of model predictions likely applies across diverse taxa and geographic regions. Because hydrilla is a notorious aquatic invasive species (Langeland 1996), occurrences are uniquely well documented around the world. Thus, removing some data caused only modest changes in model accuracy because remaining data were still numerous and widespread. Previous studies have demonstrated that Maxent possesses high predictive capacity even at small (*N* < 30) sample sizes (Wisz et al. 2008). It is likely that spatial biases like those imposed in our experiment would much more dramatically impact predictive strength of models for species with fewer documented occurrences or poorly understood ranges.

Our study contributes to the growing understanding of how spatial biases in species occurrence records can impact species distribution model predictions. Regardless of focal species, we recommend that future species distribution modeling efforts begin with a reflection on potential spatial biases of available occurrence data. If data are abundant and evenly dispersed across a species range, absences in reporting from individual countries or ecoregions represent limited challenges to the robust Maxent modeling framework. However, in situations where data are limited or spatially aggregated, conspicuous omissions of political boundaries or ecoregions can have greater influence on model predictions. Inaccurate models of species distributions can in turn hamper management efforts for invasive species, imperiled species, or other species of interest. The sampling intensity and accuracy of occurrence data used for training models represent important considerations for all species distribution modeling efforts, especially when models will be used to extrapolate beyond the native range into potentially invasible habitat. Improved biodiversity surveillance and reporting will provide great benefit not only in invaded ranges but within underreported and unexplored native ranges as well.

## Supporting Information

**Appendix S1.** Description of original *Hydrilla verticillata* occurrence data collection methods. **Appendix S2** List of global *Hydrilla verticillata* occurrence records. **Appendix S3.** Methods and results from pilot tests of Maxent regularization parameter. **Figure S3**. Evaluations of different regularization multipliers within Maxent models of *Hydrilla verticillata* habitat in its native range. **Appendix S5.** MESS and MOP outputs from Maxent model runs. **Figure S5.1.** Multivariate environmental similarity surfaces (MESS; Elith et al. 2010) output generated by Maxent. **Figure S5.2.** Mobility-oriented parity (MOP; Owens et al. 2013) output generated by Maxent. **Appendix S6.** Methods and results of bias grid implementation. **Figure S6.1.** Projection of suitable *Hydrilla verticillata* habitat in North America based on separate Maxent models developed with all native range data or native range data excluding occurrences from Australia, China, Japan, South Korea, or Thailand. **Figure S6.2.** Comparison of AUCs for models used to predict *H. verticillata* occurrence in North America, but trained on different subsets of *H. verticillata* occurrence data from the native range. **Data S1.** Data used in Appendix S3 (Methods and results from pilot tests of Maxent regularization parameter).
