# Supplementary material for: Geographic selection bias of occurrence data influences transferability of invasive Hydrilla verticillata distribution models
Source: Ecol Evol. 2014 May 26;4(12):2584–93. doi: 10.1002/ece3.1120 (PMC4203300; doi:10.1002/ece3.1120)
Supplement: Supplementary file 1 [file ece30004-2584-sd1.docx]

**Appendix S1:** Description of original *Hydrilla verticillata* occurrence data collection methods

**Native Range Survey**

Hydrilla occurrence was recorded during wide-ranging surveys throughout its native range to find biological control agents of this serious aquatic weed to be used in the invasive range in the USA. Beginning in the 1980’s, surveys have been conducted by the USDA Agricultural Research Service in conjunction with Asian collaborators, and surveys continue to the present day. As part of this research, Hydrilla collections were made in Australia, China, Indonesia, Malaysia, Singapore, and Thailand. Hydrilla was usually collected by hand from shore or while wading. At inaccessible locations, a rake head attached to a rope was used. In deeper water, a boat was utilized to reach the hydrilla.

**Ohio River Survey**

Between June and September 2010, we surveyed for invasive macrophytes at public boat launches along the Ohio River and its tributaries in Indiana, Ohio, and Illinois. Macrophytes within approximately 10 m of the shore were collected via rake toss, using either two 16 inch rake heads tied back to back or 36 inch wide standard plant rake. Rake tosses occurred at 10 m increments over a total of 50 m from both sides of each boat launch ramp. We visually surveyed boat launches in which rip rap or other obstruction precluded rake use.

**Appendix S3:** Methods and results from pilot tests of Maxent regularization parameter

To identify an appropriate regularization parameter (β) for use in Maxent modeling with our hydrilla occurrence data set, we performed a pilot tuning experiment following the example of Radosavljevic & Anderson (2013). Using rarified native range occurrence data (see main text), we developed and tested models with *k*-fold cross validation (*k* = 10) for a range of possible regularization parameter values (β = 0.25, 0.5, 1, 1.5, 2, 4, 6, 8, or 10). As a threshold-independent quantitative assessment of the effect of regularization parameter on model performance, we calculated the difference between training and testing AUC values (Warren & Seifert 2011). As a threshold-dependent assessment, we examined the 10 percentile training omission threshold calculated within the Maxent software (Radosavljevic & Anderson 2013).

Regardless of regularization parameter value, all models demonstrated strong predictive performance (AUC > 0.8), and high transferability between models based on data subsets within the native range justified our larger experimental design assessing model transferability between native and North American ranges. The difference between training and testing data AUC values was largest when regularization parameter values were smallest, and this difference decreased as regularization parameter increased (Fig. S3). The most precipitous decrease in difference between training and testing data AUC values occurred between regularization values of β = 0.25 and β = 1, and the change in values with increasing regularization parameters leveled off at values larger than β = 1. Ten percentile training omission rate also demonstrated its steepest decline between β = 0.25 and β = 1, but it declined further for β = 1.5 before increasing slightly and leveling off (Fig. S3). Considering both quantitative assessments of the effect of regularization parameter on model performance, we concluded that the default regularization parameter within the Maxent software (β = 1) represented a reasonable value for use with our data.


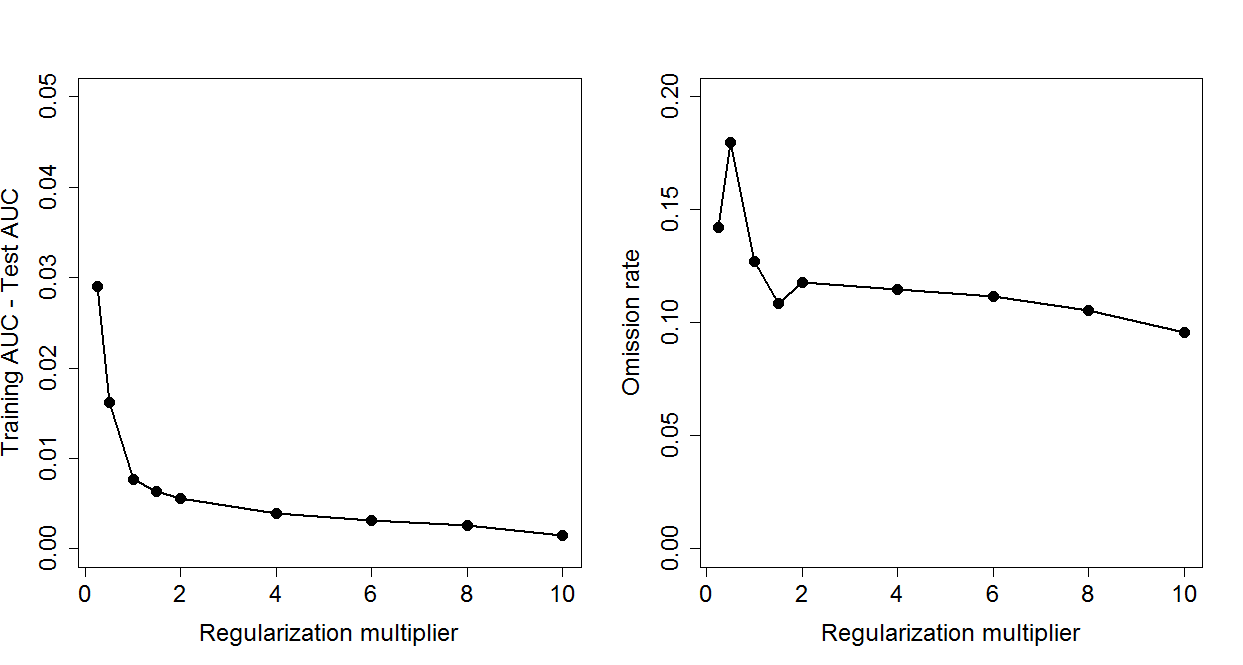


Figure S3: Evaluations of different regularization multipliers within Maxent models of *Hydrilla verticillata* habitat in its native range. Data represent the average across ten-fold iterations. Left: training AUC minus testing AUC. Right: omission rate using the 10^th^ percentile presence threshold.

**Appendix S5:** MESS and MOP outputs from Maxent model runs


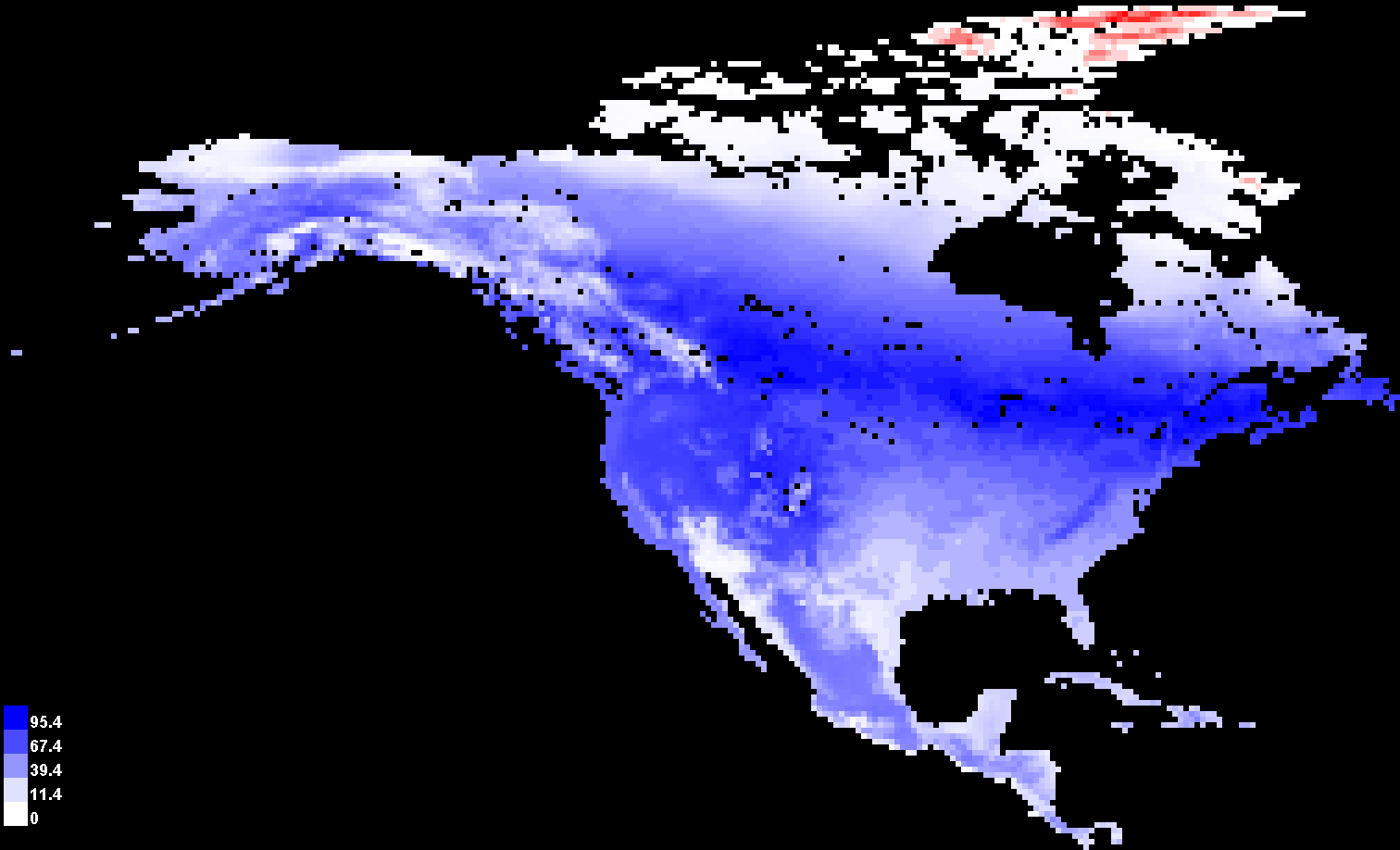


Figure S5.1. Multivariate environmental similarity surfaces (MESS; Elith et al. 2010) output generated by Maxent. Red shading in northernmost Nunavut, Canada indicates areas within North America in which one or more environmental variables have values outside the range found within the native range training data.


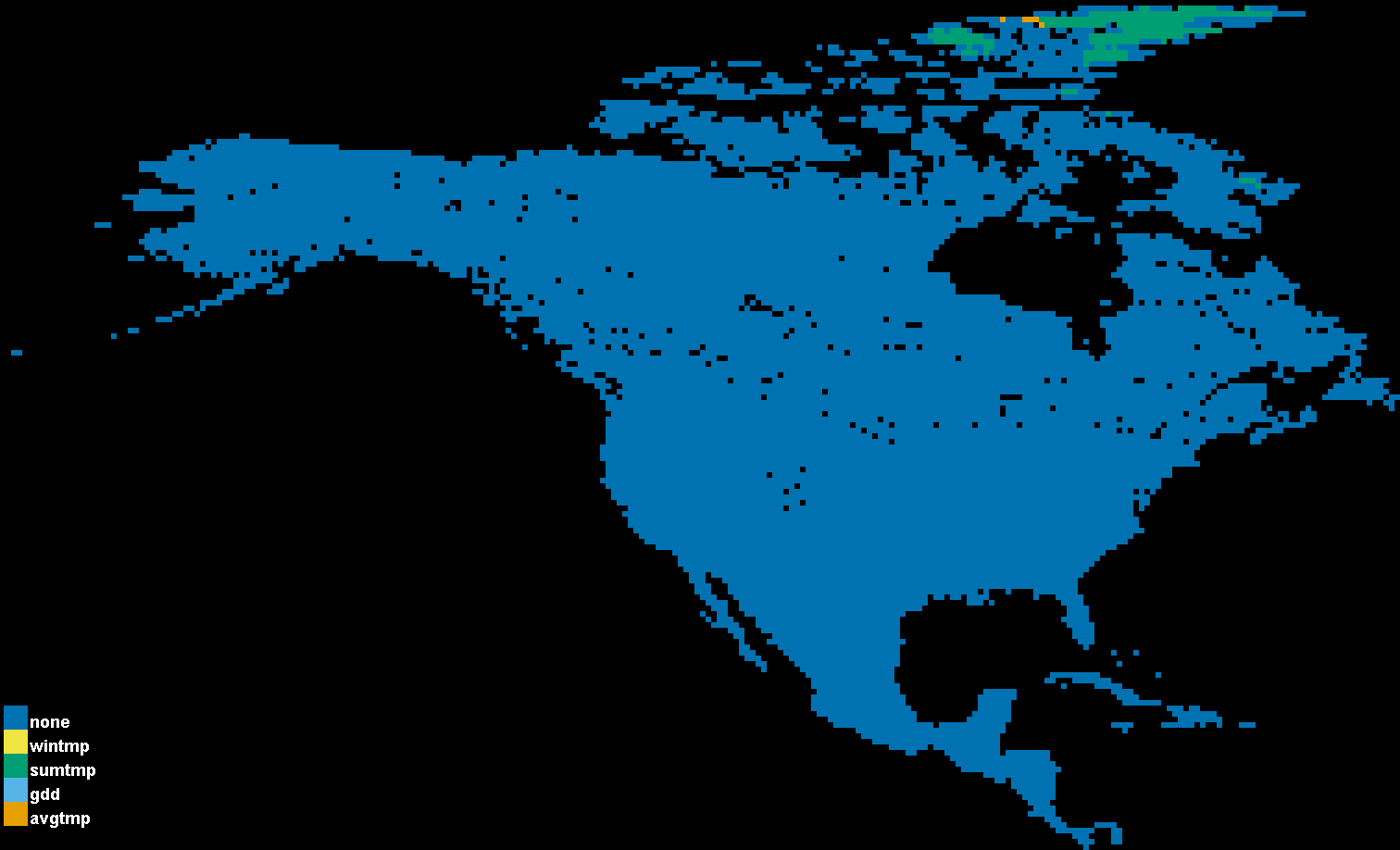


Figure S5.2. Mobility-oriented parity (MOP; Owens et al. 2013) output generated by Maxent. Shading indicates which environmental variable extended furthest beyond the range represented by native occurrence data (green = summer temperature and orange = average temperature).

**Appendix S6:** Methods and results of bias grid implementation

Bias grids prompt Maxent to weigh the importance of occurrence records inversely proportional to their proximity to neighboring occurrences, so they represent one potential option for correcting underlying sampling biases in species occurrence data (Elith *et al.* 2010, Kramer-Schadt *et al.* 2013). We developed a bias grid for the entire native range hydrilla occurrence data set as well unique bias grids for each data set representing exclusion of occurrences based on political boundaries (i.e. models excluding data from Australia, China, Japan, South Korea, or Thailand) using methods adapted from Tingley and Clements (available online: <http://dl.dropboxusercontent.com/u/11171634/Rimba_toolbox/Bias_grid_instructions.pdf>).

We used rarified occurrence data (see main text), and background data included all countries in which hydrilla occurrences are known as well as the countries that border them in Asia and Australia, coinciding with general descriptions of hydrilla native range (Cook & Lüönd 1982, Buckingham & Bennett 1996). Rarified occurrence data began in point file format, and we created a point feature layer of background data by converting our raster dataset of growing degree days in the native range to a point features (all bias grid development and visualization was performed in ArcGIS 10.1, Environmental Systems Research Institute, Redlands, California, USA).

We projected occurrence and background point datasets to World Equidistant Conic projection, and then we used Geospatial Modelling Environment version 0.7.2.1 (Spatial Ecology, LLC) to calculate distance between each occurrence and background point. We calculated the Gaussian weight of each background point in SPSS version 22 (IBM Corporation) using the equation

$$e^{(-(d^{2})/(2 \times s^{2}))}$$

where d = distance between points and s = standard deviation of the occurrence data. We selected 55 km as our standard deviation, reflecting the size of one cell in our environmental data. We joined aggregated Gaussian weight data with our original background point dataset in ArcGIS to create a bias grid shapefile and projected its datum to WGS84 to match the environmental layers used in Maxent.

To determine whether bias grids could improve model performance when occurrence data contain biases due to missing data based on political boundaries, we repeated the experiment reported in the main text while including bias grids in model development. Briefly, we prepared one Maxent model using all available native range hydrilla occurrences to predict suitable habitat in North America. We compared this model with five models using native occurrences as the training data set but omitting a different country within the native range and using a bias grid developed with the limited data set. We produced 10 additional models for each country in which an equally sized random sample of occurrence data were omitted from across the native range, using the initial all-data bias grid within each model. We projected logistic output from each Maxent model run in North America for qualitative comparison, and we calculated area under the receiver operating characteristic curve (AUC) of each model based on rarified North American hydrilla occurrence records and a constant set of randomly generated pseudo-absence data from across North America (see main text).

In general, visual inspection of output maps indicated that the extent of predicted suitable hydrilla distribution, the distribution of areas of relatively high and relatively low habitat suitability, and the qualitative differences between models excluding different occurrence data did not differ between experiments without bias grids (main text Fig. 3) and experiments in which bias grids were employed (Fig. S6.1). Quantitative comparisons also produced similar results between experiments with (Fig. S6.2) and without (main text Fig. 4) bias grids. The inclusion of bias grids slightly reduced AUC across all models. In both cases with and without bias grids, omitting data based on political boundaries resulted in larger shifts in model accuracy than omitting randomly selected occurrence data.

**
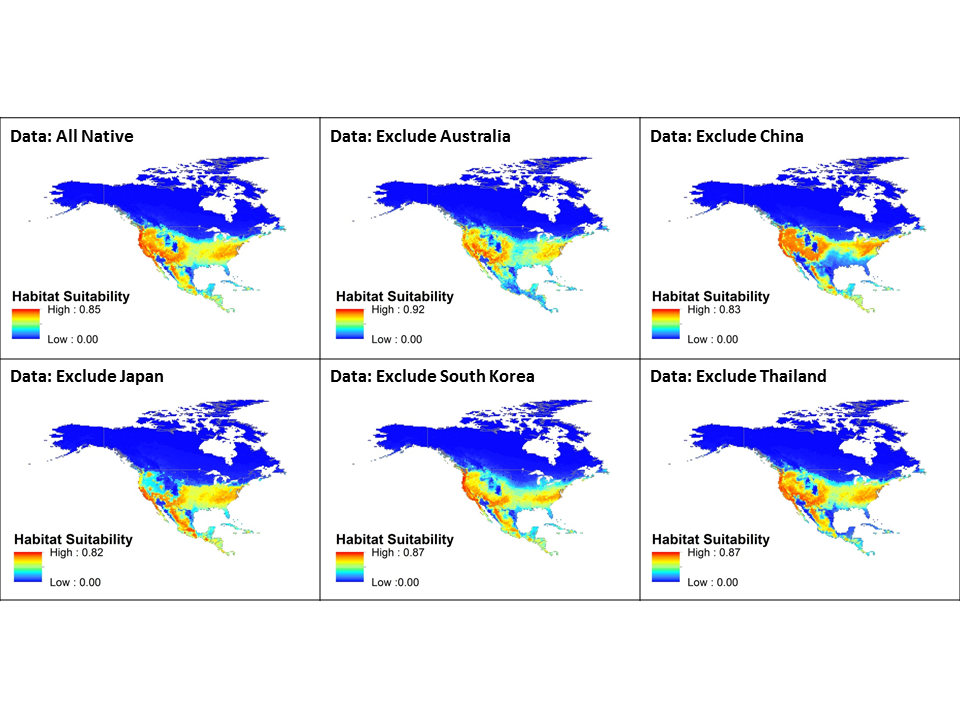
**

Figure S6.1 Projection of suitable *Hydrilla verticillata* habitat in North America based on separate Maxent models developed with all native range data or native range data excluding occurrences from Australia, China, Japan, South Korea, or Thailand. Shading indicates the logistic output of each model


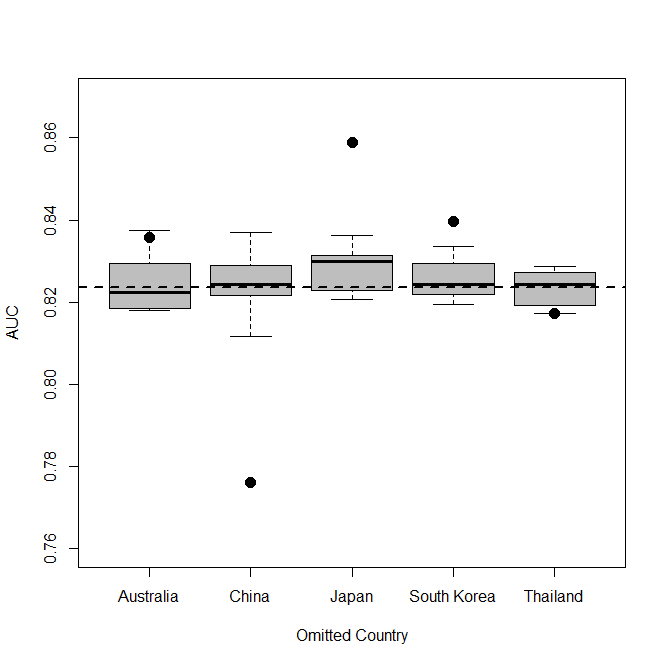


Figure S6.2 Comparison of AUCs for models used to predict H. verticillata occurrence in North America, but trained on different subsets of H. verticillata occurrence data from the native range. Dashed horizontal line indicates AUC (=0.8237) calculated for the model developed using all native range data (i.e. all countries’ occurrence records included), and filled circles represent AUCs for models trained with native data from which occurrences within specific countries were excluded. Box-and-whisker plots represent 10 models developed for each country with an equal number of randomly selected data omitted from across the native range.
